# Proteomic Analysis of Gossypol Induces Necrosis in Multiple Myeloma Cells

**DOI:** 10.1155/2014/839232

**Published:** 2014-08-14

**Authors:** Renhua Xu, Enbing Tian, Haiping Tang, Chongdong Liu, Qingtao Wang

**Affiliations:** ^1^Clinical Laboratory of Beijing Chaoyang Hospital, Capital Medical University, Beijing 100020, China; ^2^Binzhou Medical University, Yantai 264003, China; ^3^Department of Nuclear Medicine, Dongzhimen Hospital, Beijing University of Chinese Medicine, Beijing 100700, China; ^4^School of Life Sciences, Tsinghua University, Beijing 100084, China

## Abstract

Gossypol is a phenolic aldehyde extracted from plants and is known to be an antitumor agent to induce cancer cell apoptosis. In the present study, multiple myeloma cells were treated with gossypol, which resulted in an increase of cellular reactive oxygen species (ROS) and cell necrosis. Quantitative proteomic analysis was carried out to identify differentially expressed proteins between untreated and gossypol-treated cells. Proteomic analysis identified 4330 proteins, in which 202 proteins are upregulated and 383 proteins are downregulated in gossypol-treated cells as compared to the untreated cells. Importantly, proteomic and western blot analysis showed that apoptosis regulators BAK and Bax were upregulated in gossypol-treated cells, indicating that Bcl-2 associated death pathway was activated. Similarly, gossypol also induced upregulations of DNA mismatch repair proteins and DNA replication licensing factor, suggesting that gossypol caused significant DNA damage. Furthermore, upregulations of HLA class I and class II histocompatibility antigens and beta-2-microglobulin were observed in gossypol-treated cells, indicating that gossypol has a novel function to activate cellular immune responses. Our data demonstrate that the execution of necrosis is a complex process involving ROS, DNA damage, and Bcl-2 family proteins. Gossypol-activated immune responses are a potential new approach for multiple myeloma chemotherapy.

## 1. Introduction

Multiple myeloma (MM) is a clonal B-cell disorder in which malignant plasma cells (PC) accumulate in the bone marrow, resulting in lytic bone lesions and excessive amounts of monoclonal proteins. It accounts for 10% of hematologic malignancies [1]. The genomic character of MM is the chromosome translocations via juxtaposition of a set of genes to the immunoglobulin heavy chain locus, which results in overexpression of the translocalized genes such as CCND1, CCND3, MAF, MAFB, MMSET, and FGFR3 [2]. Mutations in NRAS, KRAS, FAM46C, DIS3, TP53, CCND1, PNRC1, ALOX12B, HLA-A, and MAGED1 are frequently observed in MM patients [3]. Activation of MYC, FGFR3, KRAS, and NRAS and the NF-*κ*B pathway play important roles in tumor progression [2–5]. It has been shown that abnormal methylation of tumor suppressor genes is a common event in malignant plasma cell disorders [6–8] and aberrant global methylation patterns also affect the molecular pathogenesis of MM [7]. Studies also show that microRNAs are important for the initiation and progression of MM [9–12]. Although therapeutic interventions have been developed and the overall survival has been improved over the last decade [13], MM is still incurable and most MM patients who survive initial treatment will develop drug resistance and eventually relapse. Development of new therapeutic interventions is strikingly needed for increasing patient survival rate.

Gossypol, a phenolic aldehyde extracted from the cotton and the tropical plants, can permeate cells. Gossypol forms an extensive hydrogen bonding network with residues Arg146 and Asn143 in Bcl-2 through the aldehyde group and the adjacent hydroxyl group on the right naphthalene ring [14]. Gossypol has been identified as a BH3-mimetic (BH3 stands for Bcl-2 homology domain 3) inhibitors of antiapoptotic Bcl-2 family members, including Bcl-2, Bcl-xL, and Mcl-1, and induces apoptosis in various types of cancer [15–18]. Gossypol is now in phases II and IIb clinical trials for hormone-refractory prostate cancer and other types of cancer including lung cancer, breast cancer, and ovarian cancer with promising initial results [19, 20]. Besides its binding to Bcl-2 proteins, gossypol or its derivatives have been found to act as inhibitors for several dehydrogenase enzymes [21, 22], in which they compete with NADH for binding to lactate dehydrogenase and inhibition of lactate dehydrogenase activity. Gossypol also mediates many signaling pathways, such as inhibition of the growth of prostate cancer cells by modulation of TGF-beta/Akt signaling pathway [23] and activation of TP53 [24] and enhancement of radiation-induced apoptosis through SAPK/JNK pathway [25]; apogossypolone (ApoG2), a novel derivate of gossypol, suppresses the c-Myc signaling pathway [26]; gossypol also suppresses NF-kappaB activity and NF-kappaB-related gene expression in human leukemia U937 cells [27]. It also reversibly inhibits calcineurin and binds to calmodulin. Gossypol and its enantiomer (AT-101) can regulate expressions of proangiogenic molecules released from cancer cells, suggesting the potential role in antiangiogenesis through modulating VEGF signaling-mediated angiogenesis [28, 29].

Studies further showed that gossypol affects ROS-dependent mitochondria, activates death receptor 5 pathway, and reduces tumor growth through oxidative stress in human colorectal carcinoma cells [30–32]. Oxidative stress is involved in diverse signaling pathways in cells and regulates a series of biological processes including cell growth, differentiation, proliferation, apoptosis, and even intercellular communication. Excessive oxidative stress results in damage to all the important cellular components like proteins, DNA, and membrane lipids which can cause cell death. Many drugs utilize the mechanism of oxidative stress to achieve their respective therapeutic purposes [33–35].

A few studies have been reported on the comprehensive analysis of gossypol-induced changes in cell proteome [36, 37]. It was found that oxidative phosphorylation-related proteins were depressed and endoplasmic reticulum stress markers were upregulated combination in gossypol-treated cells. The gossypol-induced mitochondrial stress was further enhanced when cells were further treated with valproic acid with upregulation of glycolysis- and hypoxia-associated proteins and downregulation of DNA damage repair proteins. Metabolomic and redox proteomic analysis of untreated and gossypol-treated ovarian cancer cells showed that gossypol induces decrease of the cellular GSH, aspartic acid, and FAD and identified changes in the thiol-redox states of 545 cysteine-containing peptides from 356 proteins [37]. In the present work, we show that gossypol-treatment enhances cellular ROS production and induces necrosis of multiple myeloma cells. Importantly, we have found that gossypol-treatment induces upregulations of molecules associated with antigen presentation, suggesting that gossypol has a novel function to activate cellular immune responses.

## 2. Materials and Methods

### 2.1. Chemicals and Reagents

RPMI-1640 medium, phosphate-buffered saline (PBS), and fetal bovine serum (FBS) were purchased from Wisent (Montreal, QC) and used without further purification. Penicillin/streptomycin and normal and dialyzed fetal bovine serum (FBS and D-FBS) were purchased from Wisent (Montreal, QC). SILAC RPMI-1640 medium, isotope labeling L-^13^C_6_Arginine*·*HCl, standard L-Arginine*·*HCl, isotope labeling L-^13^C_6_^15^N_2_ Lysine*·*HCl, standard L-Lysine*·*HCl, and mass spectrum grade acetonitrile were purchased from Thermo (Waltham, MA). Dithiothreitol (DTT) was purchased from Merck (Whitehouse Station, NJ). Pronase E was purchased from Roche (South San Francisco, CA). Sequencing grade trypsin was purchased from Promega (Fitchburg, WI). Gossypol, iodoacetamide (IAA), and RNase A were purchased from Sigma (St. Louis, MO). Dimethyl sulfoxide (DMSO) was purchased from Applichem (St. Louis, MO). A BCA protein assay kit was purchased from Solarbio (Tongzhou District, Beijing). Anti-Bcl-2, Anti-Bax, and Anti-HLA-A antibodies were purchased from Abcam (Cambridge, MA). Anti-HLA-DR antibody was purchased from Biolegend (California, USA). Anti-NDUFA5 antibody was purchased from Pierce (Rockford, IL). Anti-HSP60 antibody was purchased from Stressgen (Victoria, BC). Anti-P53 antibody was purchased from Sigma (St. Louis, MO). Anti-beta-actin antibody was purchased from Abmart (Xuhuidistrict, Shanghai). Anti-caspase-3 antibody, anti-mouse secondary antibody, and anti-rabbit secondary antibody were purchased from Cell Signaling Technology (Beverly, MA). The cell lysis buffer for Western and BCA protein assay kit was purchased from Biyuntian (Haimen, Jiangsu, CN). HBSS was purchased from Gibco (Grand Island, NY). Protease Inhibitor Cocktail was purchased from Pierce (Rockford, IL). Image-iT LIVE Green Reactive Oxygen Species (ROS) Detection Kit was purchased from Invitrogen Molecular Probes (Eugene, OR). The kit provides the assay based on 5-(and-6)-carboxy-2′,7′-dichlorodihydrofluorescein diacetate (carboxy-H_2_DCFDA), a reliable fluorogenic marker for ROS in live cells.

### 2.2. Cell Culture and Sample Preparation

Human MM cell line U266 was purchased from the Tumor Cell Bank of Chinese Academy of Medical Sciences (Beijing, China). Cells were cultured in RPMI-1640 containing 10% or 15% FBS with 1% penicillin/streptomycin at 37°C in a humidified incubator with 5% CO_2_. For SILAC labeling, cells were washed with PBS prior to being cultured in SILAC culture medium, which was made by mixing SILAC RPMI-1640 medium with 10% D-FBS, 1% penicillin/streptomycin, 200 mg/L isotope labeling L-^13^C_6_ Arginine*·*HCl, and 40 mg/L isotope labeling L-^13^C_6_^5^N_2_ Lysine*·*HCl. U266 cells were grown for 8 to 10 passages in SILAC medium and tested for full incorporation. Prior to treatment, cells were cultured for at least 12 h to reach exponential growth phase. Cells were treated with gossypol dissolved in DMSO and the control cells were treated with the same amount of DMSO for the same time periods. After treatments, cells were washed twice with ice-cold PBS and lysed with RIPA lysis buffer (25 mmol/L Tris-HCl pH 7.6, 150 mmol/L NaCl, 0.1% SDS, 1% NP-40, 1% sodium deoxycholate, 1 mmol/L PMSF, and Roche Complete Protease Inhibitor Cocktail) for 30 min on ice. Cell lysates were clarified by centrifugation at 14,000 ×g for 20 min at 4°C. The protein concentration in the supernatant of each sample was determined using a BCA protein assay kit.

### 2.3. Protein Separation by 1D SDS-PAGE and Proteomic Analysis

Equal amounts of proteins from untreated and gossypol-treated U266 cells were mixed together and separated by 1D SDS-PAGE. The gel bands were excised from the gel into 10 slices, reduced with 25 mmol/L DTT, and alkylated with 55 mmol/L IAA. In-gel digestion was then carried out with sequencing grade trypsin in 40 mmol/L ammonium bicarbonate at 37°C overnight. The peptides were extracted twice with 0.1% formic acid in 50% acetonitrile aqueous solution for 30 min. Extracts were then centrifuged in a Speedvac to reduce the volume.

For LC-MS/MS analysis, the digestion product was separated by a 120 min gradient elution at a flow rate 0.250 *μ*L/min with an EASY-nLCII integrated nano-HPLC system (Proxeon, Denmark) which was directly interfaced with a Thermo LTQ-Orbitrap mass spectrometer. The analytical column was a home-made fused silica capillary column (75 *μ*m, 150 mm length; Upchurch, Oak Harbor, USA) packed with C-18 resin (300 Å, 5 *μ*m, Varian, Lexington, USA). Mobile phase A consisted of 0.1% formic acid aqueous solution, and mobile phase B consisted of 99.9% acetonitrile and 0.1% formic acid. The LTQ-Orbitrap mass spectrometer was operated in the data-dependent acquisition mode using Xcalibur 2.0.7 software and there was a single full-scan mass spectrum in the orbitrap (300–1,800 m/z, 70,000 resolution) followed by 20 data-dependent MS/MS scans in the ion trap at 35% normalized collision energy (CID).

The MS/MS spectra from each LC-MS/MS run were searched against the human protein database from UniProt (release date of March 192,014; 68,406 entries) using an in-house Proteome Discoverer (Version PD 1.4, Thermo-Fisher Scientific, USA).The search criteria were as follows: full tryptic specificity was required; one missed cleavage was allowed; carbamidomethylation was set as the fixed modification; the oxidation (M) and SILAC ^13^C_6_ Arginine (+6.02013 Da at arginine) and SILAC ^13^C_6_^15^N_2_ Lysine (+8.01420 Da at lysine) were set as the variable modification; precursor ion mass tolerances were set at 10 ppm for all MS acquired in an orbitrap mass analyzer; and the fragment ion mass tolerance was set at 0.6 Da for all MS2 spectra acquired in the linear ion trap. An identified peptide with a *q* value <0.01 was considered as a positive identification. Database searching against the corresponding decoy database was also performed to evaluate the false discovery rate (FDR) of peptide identification.

Protein quantitation was also carried out with Proteome Discoverer Searching Algorithm (Version 1.4). Briefly, ratios of relative protein expressions for each arginine- or lysine-containing peptide were calculated using the peak area of Arg6 or Lys8 divided by the peak area of Arg0 or Lys0. The protein ratio is then calculated by averaging all peptide ratios for that protein. Quantitative precision was expressed as protein ratio variability.

### 2.4. DNA Fragment Assay

DNA fragment assay was performed following the procedure described by Mazars et al. [38]. Briefly, cells were washed with PBS twice and collected by centrifugation. Cells were suspended in 250 *μ*L lysis buffer (1% NP-40, 20 mmol/L EDTA, 50 mmol/L Tris-HCl, pH 7.5). The supernatants were collected by centrifugation for 5 min at 1,600 ×g. The supernatant was incubated with 0.71 mg/mL RNase A for 2 h at 56°C. Then, 100 *μ*g/mL pronase E was added and incubated with the supernatants overnight at 37°C. DNA fragments were precipitated with 0.5 volumes of 10 mol/L ammonium acetate and 2 volumes of ethanol at −20°C for 12 h and centrifugation for 15 min at 15,000 ×g. The precipitate was washed with 70% ethanol and resuspended in loading buffer. Electrophoresis was performed in 0.5x Tris-borate-EDTA buffer for 30 min.

### 2.5. Flow Cytometry

Cells were treated with gossypol at different concentrations. After treatments, cells were harvested and spun down at 1,000 ×g for 5 min. The medium was discarded and washed with PBS twice. Next, cells were resuspended in 1x annexin V binding buffer supplied with the Apoptosis and Necrosis Quantification kit. Then, 5 *μ*L of FITC-annexin V and 5 *μ*L of ethidium homodimer III solutions were added to each tube. After incubation at room temperature for 15 min in the dark, 400 *μ*L 1x binding buffer was added to each tube and the cells were analyzed with a BD FACSCalibur Flow Cytometer (FACSCalibur, BD Biosciences, San Jose, CA) within 1 h of staining. Dot plots and histograms were analyzed by CellQuest Pro software (BD Biosciences, Heidelberg, Germany).

### 2.6. Western Blot Analysis

Untreated and gossypol-treated cells were collected and lysed on ice with Biyuntian cell lysis buffer containing 20 mmol/L Tris (pH 7.5), 150 mmol/L NaCl, 1% Triton X-100, and sodium pyrophosphate, *β*-glycerophosphate, EDTA, and Na_3_VO_4_ for Western Blotting and IP supplied with the Protease Inhibitor Cocktail. The supernatants were collected after centrifugation at 14,000 ×g for 10 min at 4°C. Protein concentrations were determined using the BCA protein assay kit. Proteins were separated on a 12% SDS-PAGE gel and transferred onto a PVDF transfer membrane by electroblotting. After blocking with 5% nonfat milk for 2 h at room temperature, the membrane was incubated overnight at 4°C with 1,000x diluted primary antibody, washed with PBST buffer for 3 times, and then incubated with 1,000x diluted anti-mouse or anti-rabbit secondary antibody labeled with HRP at room temperature for 2 h. The membrane was further washed with PBST buffer 3 times and developed using the Enlighten Kit (Engreen, China). Beta-actin was detected with anti-actin antibody as an internal control.

### 2.7. Detection of Reactive Oxygen Species (ROS) in Untreated and Gossypol-Treated Cells

ROS in untreated and gossypol-treated cells was detected using an Image-iT LIVE Green Reactive Oxygen Species Detection Kit following the manufacturer's instructions. Briefly, the cells were collected by centrifugation and washed once with warm HBSS/Ca/Mg. Cells were resuspended with 500 *μ*L of the 25 *μ*mol/L carboxy-H_2_DCFDA working solution for 25 min at 37°C, followed by addition of the Hoechst 33342 reagent to the reaction mixture at a final concentration of 1.0 *μ*mol/L and incubation for 5 min. The final products were washed gently with 1 mL HBSS/Ca/Mg immediately followed by imaging with LSM780 Confocal Microscopy.

### 2.8. Statistics Methods

Results are expressed as the mean ± SD. Student's *t*-test was used for comparisons between untreated and gossypol-treated cells. A value of *P* < 0.05 was considered as statistically significant. All analyses were conducted using the SPSS 17.0 software (SPSS Inc, Chicago III).

## 3. Results

### 3.1. Gossypol Enhances ROS Production and Induces Multiple Myeloma Cell Necrosis

**Figure 1:**
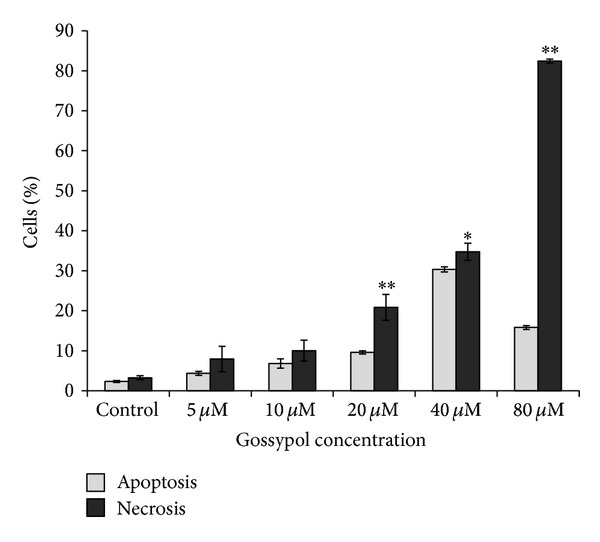
Percentage of necrosis-related cell death in U266 cells treated with gossypol (0–80 *μ*mol/L) for 24 h. Results are expressed as the mean of three repeats. Significant necrosis was observed with 20 *μ*mol/L gossypol-treatment. ∗ for *P* < 0.05 and ∗∗ for *P* < 0.01.

**Figure 2:**
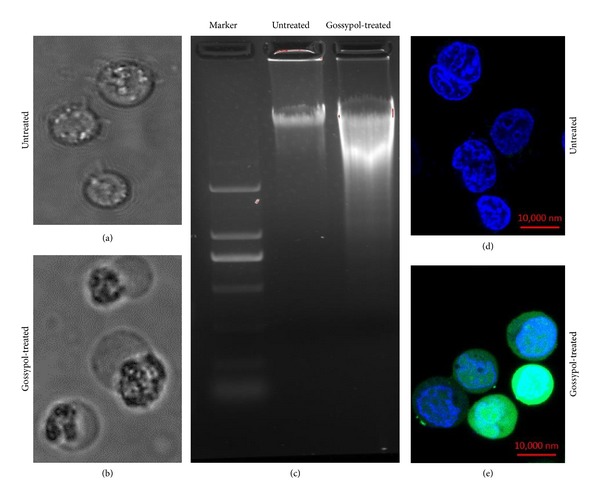
Morphologic images of multiple myeloma cells. (a) Untreated cells; (b) 40 *μ*mol/L gossypol-treated cells for 24 h; all images were captured by Olympus IX2-UCB 60x inverted microscopy; (c) gel electrophoresis of DNA from untreated and gossypol-treated myeloma cells. (d-e) Detection of ROS in untreated and gossypol-treated U266 cells using the Image-iT LIVE Reactive Oxygen Species (ROS) Kit. Cells were labeled with carboxy-H_2_DCFDA, which exhibited green fluorescence when reacted with ROS, and nuclei were stained with blue-fluorescent Hoechst 33342. (d) Fluorescence image of ROS in untreated U266 cells; and (e) fluorescence image of ROS in 40 *μ*mol/L gossypol-treated cells for 10 h.

FACS analysis showed that the percentage of necrotic cells was 22% when cells were treated with 20 *μ*mol/L gossypol for 24 h, increasing to 82% when treated with 80 *μ*mol/L gossypol for 24 h (Figure 1). Morphological features of the dying cells were consistent with the cell necrosis. Images of cell morphology in untreated and gossypol-treated cells are shown in Figures 2(a) and 2(b), respectively. The gossypol-treated multiple myeloma cells displayed characteristic features of necrosis, including cell swelling, translucent cytoplasm, cell membrane disruption, pyknotic nuclei, and excessive cellular debris. The DNA content of necrotic cells was analyzed by gel electrophoresis. The gel image of DNA for untreated and gossypol-treated cells (Figure 2(c)) shows that DNA from gossypol-treated cells exhibited a random and general cleavage pattern and produced a smear that further confirmed that gossypol-induced cell death occurs mainly via necrosis. The above data suggests that oxidative stress may cause necrosis in gossypol-treated cells. To confirm that ROS contributes to gossypol-induced cell necrosis, an Image-iT LIVE Reactive Oxygen Species (ROS) Kit was used to detect ROS in the untreated and gossypol-treated cells. Cells were labeled with carboxy-H_2_DCFDA, which fluoresces when oxidized by ROS, and nuclei were stained with blue-fluorescent Hoechst 33342. The gossypol-treated cells exhibited much stronger green fluorescence (Figure 2(e)) in comparison to untreated cells (Figure 2(d)), indicating that gossypol induced a significant increase in ROS production.

### 3.2. Proteomic Analysis of Gossypol-Treated Multiple Myeloma Cells

**Figure 3:**
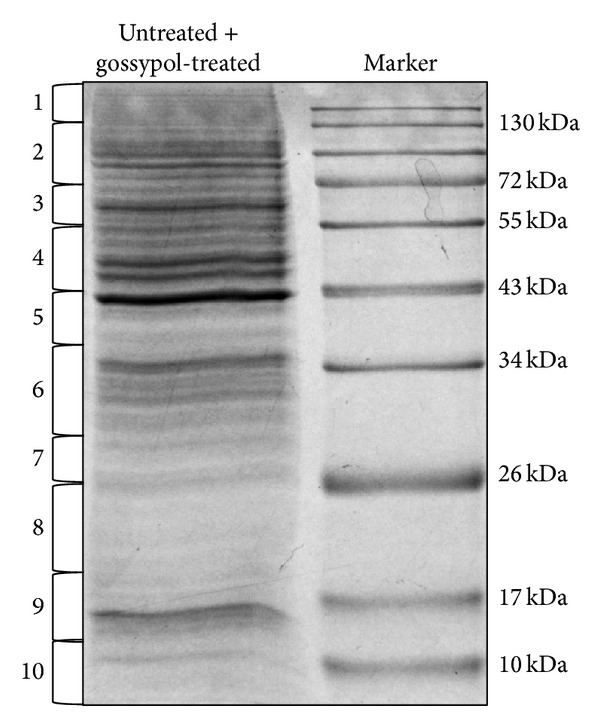
The 1D SDS-PAGE gel image of proteins from untreated and 24 h, 40 *μ*mol/L gossypol-treated multiple myeloma cells. Lane 1: proteins from untreated cells cultured in the SILAC medium were mixed with proteins from gossypol-treated cells cultured in regular medium; and Lane 2: molecular weight markers.

**Figure 4:**
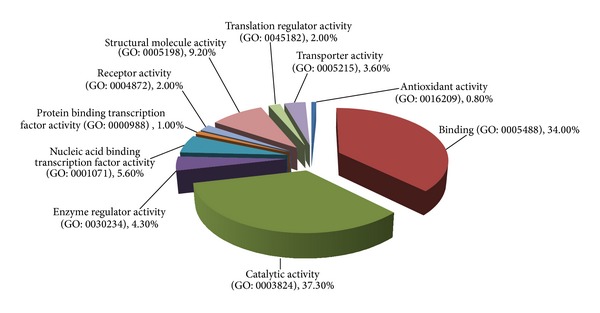
Functional classification of differentially regulated proteins with PANTHER (http://www.pantherdb.org/). The numbers of proteins related with each category are shown in brackets.

Next, proteomic analysis was carried out on the necrotic multiple myeloma cells. An equal amount of proteins (30 *μ*g) from untreated and gossypol-treated U266 cells was mixed and separated by 1D SDS-PAGE (Figure 3). Differentially expressed proteins were identified and quantified using SILAC quantitation. The experiments were repeated twice and we identified about 4330 proteins. The false-positive rate was estimated to be less than 1%. Based on SILAC ratios (>1.6 or <0.7) and the protein scores (>10), 585 proteins were found to be differentially expressed between untreated and gossypol-treated cells, in which 383 proteins are downregulated and 202 are upregulated (see Supplementary Tables 1 and 2 in Supplementary Material available online at http://dx.doi.org/10.1155/2014/839232). In order to understand the biological relevance of the identified proteins, the gene ontology (GO) was used to cluster the differentially expressed proteins according to their molecular functions and biological processes. The annotations of gene lists are summarized via a pie plot using the PANTHER bioinformatics platform (http://www.pantherdb.org/) as shown in Figure 4. 166 proteins were classified into several significant groups of biological processes according to their molecular functions including energy and carbohydrate metabolism, RNA processing and protein synthesis, DNA repair, chaperone, and cell cycle regulation.

### 3.3. Identification and Classification of Differentially Expressed Proteins

Among 202 upregulated proteins, it is noticed that DNA replication licensing factors (MCM2-7), DNA replication complex GINS proteins (PSF1, PSF3, and SLD5), and DNA mismatch repair proteins (MLH1, MSH2, and MSH6) are upregulated in gossypol-treated cells. The relative expression changes are displayed in Supplementary Figure 1. The expression level of SLD5 is 3.7 times higher in treated cells than that in untreated cells. Levels of MCM2-7 increase about 1.5-fold in treated cells as compared to untreated cells. On the other hand, it is also noticed that most mitochondrial ribosome proteins including 10 28S ribosomal proteins and 10 39S ribosomal proteins are downregulated in gossypol-treated cells as compared to the untreated cells (Supplementary Figures 2 and 3). Furthermore, many HLA class I histocompatibility antigens, HLA class II histocompatibility antigens, and beta-2-microglobulin are upregulated in gossypol-treated cells (Supplementary Table 1), indicating that gossypol activates the immune system in U266 cells. The MS/MS spectra that were used to identify HLA-A and HLA-DR were displayed in Supplementary Figure 4. Among the other upregulated proteins are the cell death associated proteins including BAK, Bcl-2-like protein 13, FAS-associated factor 1, FAS-associated factor 2, and isoform 2 of caspase-2. MS/MS spectrum for identification of BAK was displayed in Supplementary Figure 4, in which b and y fragment ions are matched to the predicted fragments from the peptide VVALLGFGYR.

### 3.4. Identification and Verification of Differentially Expressed Proteins by Western Blotting

**Figure 5:**
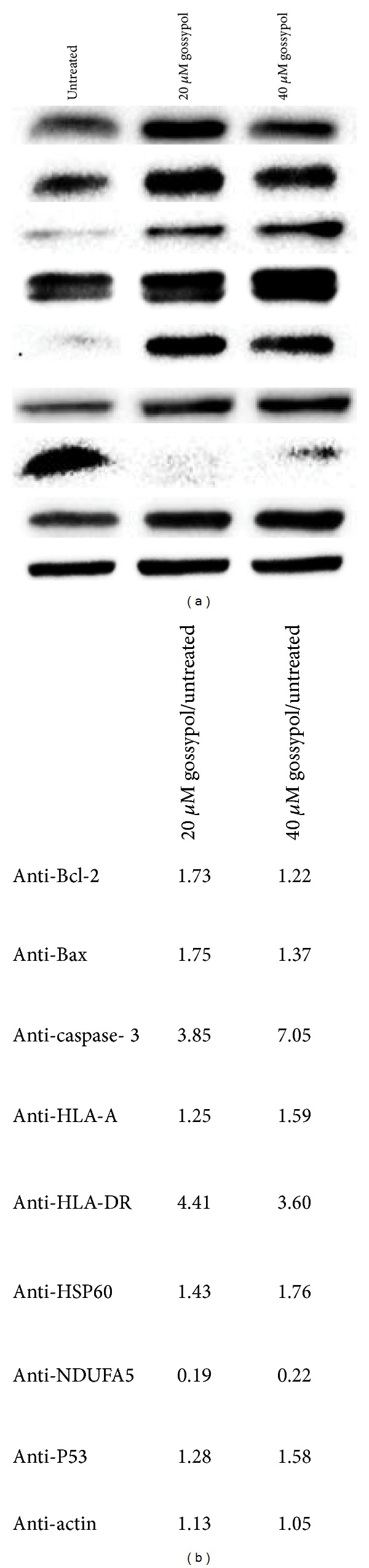
Western blot analysis of Bcl-2, Bax, caspase-3, HLA-A, HLA-DR, HSP60, NDUFA5, and P53 in untreated, 20 *µ*mol/L and 40 *µ*mol/L gossypol-treated cells. (a) Western blot image. (b) Analysis of western blots displayed in (a).

Using available quality antibodies, we carried out western blot analysis to quantify selected proteins that may play a role in gossypol-induced necrosis and to confirm selected proteins identified by proteomic analysis. Eight proteins were probed with antibodies and beta-actin was used as a control. Western blotting is displayed in Figure 5(a), showing that Bcl-2, Bax, caspase-3, HLA-A, HLA-DR, HSP60, and P53 are upregulated in gossypol-treated cells, while NADH dehydrogenase 1 alpha subcomplex 5 (NDUFA5) is downregulated. Quantitation of band intensities in western blot images was carried out using the Image Lab 4.0.1 Software (Figure 5(b)). The changes of the band intensities by western blotting are comparable to those determined by SILAC ratios for HLA-A and HLA-DR, indicating that quantitative proteomic analysis generates reliable results.

## 4. Discussions

Necrosis lacks characteristics of apoptosis and autophagy [39–43], and the occurrence and course of necrosis were found to be programmed and tightly regulated. Previous studies showed that death ligands (e.g., CD95L, TNF, and TNF-related apoptosis-inducing ligand) induced caspase-independent necrotic-like cell death that relied on the activity of the death domain- (DD-) containing kinase RIP1. The induction mechanisms of necrosis are becoming increasingly clear, but the execution of this process remains somewhat elusive. Necrosis is comprised of a complex sequence of cellular processes including mitochondrial dysfunction with enhanced generation of reactive oxygen species and ATP depletion, proteolysis by calpains and cathepsins, and early plasma membrane rupture. Studies have been reported on necrosis of multiple myeloma cells. For example, Kigamicin, a compound derived from actinomycetes, induces necrosis in human multiple myeloma cells by inhibition of cyclin D1, p21, p-AKT, and p-ERK [44]; and a D-amino acid-containing peptide HYD1 increases the reactive oxygen species (ROS) production, leading to necrotic cell death in multiple myeloma cells [45–47]. In the present study, we found that 82% cell death was attributed to necrosis when cells were treated with 80 *μ*mol/L gossypol for 24 h (Figure 1). At the same time, we have measured the cellular ROS levels and found that gossypol-treatment significantly increases cellular ROS level. Our data suggest that gossypol induces cell necrosis through oxidative stress.

To profile gossypol-induced changes in proteomes of untreated and gossypol-treated cells, we have carried out a SILAC-based protein quantitation. Among 4330 identified proteins, gossypol induced changes in expression levels of 585 proteins (Supplementary Tables 1 and 2). DNA replication associated proteins including DNA replication licensing factors (RLF) and DNA replication complex GINS are upregulated in gossypol-treated cells. RLFs acting as replicating helicases are essential to restrict the duplication of genomic DNA to precisely once per cell cycle, while the GINS complex plays an important role in the initiation of DNA replication and progression of DNA replication forks. DNA mismatch repair proteins are also upregulated in gossypol-treated cells, which initiate DNA mismatch repair by binding to a dsDNA mismatch. These results suggest that gossypol targets and damages nuclear DNA. Among the other upregulated proteins are HLA classes I and II histocompatibility antigens and beta-2-microglobulin. Upregulation of HLA-A and HLA-DR was confirmed by western blotting. HLA-I/II molecules bind to peptides derived from antigens and present them on the cell surface for activation of the immune system. The increase of HLA-I/II molecules indicates that gossypol modulates the cellular immune response system. Our proteomic and western blot analysis also show that gossypol induced the upregulation of caspase-3, Bax, and BAK (Figure 5). This is consistent with earlier reports showing that gossypol activates programmed cell death [48–50]. The mechanism underlining gossypol-mediated changes in protein expression remains elusive. Upregulations of death-associated proteins contribute to gossypol-induced necrosis.

## 5. Conclusions

Taken together, our results show that gossypol-treatment leads to increasing cellular ROS, damaging DNA, and upregulating death-associated factors including caspase-3, Bax, and BAK to induce necrosis in multiple myeloma cells. Gossypol also upregulates expression of proteins associated with antigen presenting pathways including HLA classes I and II histocompatibility antigens and beta-2-microglobulin that may elicit immune responses and be developed into a new therapeutic approach for multiple myeloma treatment.

## Supplementary Material

Supplementary Figure 1. Gossypol induced the fold of changes in DNA damage repair and replication associated proteins from the untreated and 40 *μ*M gossypol-treated U266 cells for 24 h as determined by quantitative proteomics.
Supplementary Figure 2. Gossypol induced the fold of changes in mitochondrial 28S associated proteins from the untreated and 40 *μ*M gossypol-treated U266 cells for 24 h as determined by quantitative proteomics.
Supplementary Figures 3. Gossypol induced the fold of changes in mitochondrial 39S associated proteins from the untreated and 40 *μ*M gossypol-treated U266 cells for 24 h as determined by quantitative proteomics.
Supplementary Figure 4. The MS/MS spectra of peptides for identification of selected proteins.
Supplementary Table 1. Up-regulated proteins in gossypol-treated cells.
Supplementary Table 2. Down-regulated proteins in gossypol-treated cells.
