# Supplementary material for: Proteomic Analysis of Gossypol Induces Necrosis in Multiple Myeloma Cells
Source: Biomed Res Int. 2014 Aug 14;2014:839232. doi: 10.1155/2014/839232 (PMC4150408; doi:10.1155/2014/839232)
Supplement: Supplementary file 1 — Supplementary Figure 1. Gossypol induced the fold of changes in DNA damage repair and replication associated proteins from the untreated and 40 μM gossypol-treated U266 cells for 24 h as determined by quantitative proteomics. Supplementary Figure 2. Gossypol induced the fold of changes in mitochondrial 28S associated proteins from the untreated and 40 μM gossypol-treated U266 cells for 24 h as determined by quantitative proteomics. Supplementary Figures 3. Gossypol induced the fold of changes in mitochondrial 39S associated proteins from the untreated and 40 μM gossypol-treated U266 cells for 24 h as determined by quantitative proteomics. Supplementary Figure 4. The MS/MS spectra of peptides for identification of selected proteins. Supplementary Table 1. Up-regulated proteins in gossypol-treated cells. Supplementary Table 2. Down-regulated proteins in gossypol-treated cells. [file 839232.f1.zip › Supplementary description.docx]

Supplementary Figures 1. Gossypol induced the fold of changes in DNA damage repair and replication associated proteins from the untreated and 40 µM gossypol-treated U266 cells for 24 h as determined by quantitative proteomics.

Supplementary Figure 2. Gossypol induced the fold of changes in mitochondrial 28S associated proteins from the untreated and 40 µM gossypol-treated U266 cells for 24 h as determined by quantitative proteomics.

Supplementary Figures 3. Gossypol induced the fold of changes in mitochondrial 39S associated proteins from the untreated and 40 µM gossypol-treated U266 cells for 24 h as determined by quantitative proteomics.

Supplementary Figure 4. The MS/MS spectra of peptides for identification of selected proteins. (A) MS/MS spectrum of a doubly charged peptide ion at m/z 550.8327 for MH_2_^2+^ corresponding to the mass of the peptide VVALLGFGYR from BAK; (B) The MS/MS spectrum of a doubly charged ion at m/z 565.823 for MH_2_^2+^ corresponding to the mass of the peptide VNHVTLSQPK from B2M; (C) The MS/MS spectrum of a doubly charged ion at m/z 824.40997 for MH_2_^2+^ corresponding to the mass of the peptide HKWEAAHVAEQWR from HLA-A; and (D) The MS/MS spectrum of a triply charged ion at m/z 959.165 for MH_3_^3+^ corresponding to the mass of the peptide TQTLQHHNLLVCSVNGFYPGSIEVR from HLA-DR. The labeled peaks correspond to masses of y and b ions of the peptide.

Supplementary Table 1. Up-regulated proteins in gossypol-treated cells

Supplementary Table 2. Down-regulated proteins in gossypol-treated cells
