# Supplementary material for: Proteomic Analysis of Gossypol Induces Necrosis in Multiple Myeloma Cells
Source: Biomed Res Int. 2014 Aug 14;2014:839232. doi: 10.1155/2014/839232 (PMC4150408; doi:10.1155/2014/839232)
Supplement: Supplementary file 1 — Supplementary Figure 1. Gossypol induced the fold of changes in DNA damage repair and replication associated proteins from the untreated and 40 μM gossypol-treated U266 cells for 24 h as determined by quantitative proteomics. Supplementary Figure 2. Gossypol induced the fold of changes in mitochondrial 28S associated proteins from the untreated and 40 μM gossypol-treated U266 cells for 24 h as determined by quantitative proteomics. Supplementary Figures 3. Gossypol induced the fold of changes in mitochondrial 39S associated proteins from the untreated and 40 μM gossypol-treated U266 cells for 24 h as determined by quantitative proteomics. Supplementary Figure 4. The MS/MS spectra of peptides for identification of selected proteins. Supplementary Table 1. Up-regulated proteins in gossypol-treated cells. Supplementary Table 2. Down-regulated proteins in gossypol-treated cells. [file 839232.f1.zip › Supplementary Table 2(RHX).pdf]

Supplementary Table 2. Down-regulated proteins in gossypol-treated cells

Accession	Description	Score	Coverage	Unique Peptides	Gossypol / Control
P16885	1-phosphatidylinositol 4,5-bisphosphate phosphodiesterase	135	26	26	0.7
P82912-2	28S ribosomal protein S11, mitochondrial	13	24	3	0.1
A6ND22	28S ribosomal protein S16, mitochondrial	19	26	2	0.7
E9PE17	28S ribosomal protein S17, mitochondrial	21	45	3	0.4
G5E9W7	28S ribosomal protein S22, mitochondrial	37	40	9	0.4
Q9Y3D9	28S ribosomal protein S23, mitochondrial	16	35	6	0.3
Q96EL2	28S ribosomal protein S24, mitochondrial	11	22	3	0.4
P82663	28S ribosomal protein S25, mitochondrial	12	23	4	0.4
Q9BYN8	28S ribosomal protein S26, mitochondrial	14	19	4	0.4
G5EA06	28S ribosomal protein S27, mitochondrial	17	21	5	0.5
Q9Y2Q9	28S ribosomal protein S28, mitochondrial	24	35	5	0.3
P51398-2	28S ribosomal protein S29, mitochondrial	26	20	5	0.4
P82930	28S ribosomal protein S34, mitochondrial	35	47	8	0.4
Q9Y2R9	28S ribosomal protein S7, mitochondrial	15	20	5	0.4
F8VVX6	2-methoxy-6-polyprenyl-1,4-benzoquinol methylase	12	19	3	0.7
F5H801	2-oxoglutarate dehydrogenase, mitochondrial	14	7	6	0.4
P21953	2-oxoisovalerate dehydrogenase subunit beta, mitochondrial	13	10	3	0.5
O95861	3'(2'),5'-bisphosphate nucleotidase 1	110	69	17	0.5
Q7Z7H8	39S ribosomal protein L10, mitochondrial	10	10	2	0.4
Q9Y3B7	39S ribosomal protein L11, mitochondrial	30	41	7	0.4
Q9BYD1	39S ribosomal protein L13, mitochondrial	24	49	8	0.2
Q9P015	39S ribosomal protein L15, mitochondrial	21	27	7	0.2
Q9NX20	39S ribosomal protein L16, mitochondrial	14	14	3	0.1
E9PKV2	39S ribosomal protein L17, mitochondrial	16	18	3	0.3
Q9H0U6	39S ribosomal protein L18, mitochondrial	9	18	3	0.3
S4R3W9	39S ribosomal protein L19, mitochondrial	10	21	3	0.3
I3L2X1	39S ribosomal protein L20, mitochondrial	9	32	2	0.5
Q16540	39S ribosomal protein L23, mitochondrial	12	34	4	0.2
Q13084	39S ribosomal protein L28, mitochondrial	14	29	4	0.3
P09001	39S ribosomal protein L3, mitochondrial	10	12	3	0.3
Q96DV4	39S ribosomal protein L38, mitochondrial	12	7	3	0.3
Q9H9J2	39S ribosomal protein L44, mitochondrial	17	23	5	0.4
Q9BRJ2	39S ribosomal protein L45, mitochondrial	21	17	5	0.6
Q9H2W6	39S ribosomal protein L46, mitochondrial	14	22	5	0.5
Q9HD33-2	39S ribosomal protein L47, mitochondrial	9	17	4	0.4
F5H702	39S ribosomal protein L48, mitochondrial	12	21	2	0.3
Q13405	39S ribosomal protein L49, mitochondrial	19	30	4	0.3
Q96EL3	39S ribosomal protein L53, mitochondrial	10	43	4	0.4
Q9BYD2	39S ribosomal protein L9, mitochondrial	29	33	8	0.4

Q9P035	3-hydroxyacyl-[acyl-carrier protein] dehydratase 3	20	15	4	0.7
Q99714	3-hydroxyacyl-CoA dehydrogenase type-2	256	89	17	0.7
Q9BUT1	3-hydroxybutyrate dehydrogenase type 2	31	33	7	0.5
Q96IR7	4-hydroxyphenylpyruvate dioxygenase-like protein	64	40	10	0.5
P27635	60S ribosomal protein L10	146	50	3	0.6
Q6DKI1	60S ribosomal protein L7-like 1	14	26	5	0.5
Q86V21	Acetoacetyl-CoA synthetase	10	6	4	0.6
Q13085-3	Acetyl-CoA carboxylase 1	13	3	5	0.6
Q99798	Aconitatehydratase, mitochondrial	218	52	30	0.6
P07108	Acyl-CoA-binding protein	42	64	5	0.6
O95372	Acyl-protein thioesterase 2	23	18	3	0.6
P46108	Adapter molecule crk	26	29	1	0.4
Q01518	Adenylyl cyclase-associated protein 1	100	47	23	0.7
P12235	ADP/ATP translocase 1	180	39	2	0.2
C9JZR4	ADP-ribosylation factor GTPase-activating protein 3	10	12	3	0.6
Q96BM9	ADP-ribosylation factor-like protein 8A	71	44	1	0.6
Q9Y4W6	AFG3-like protein 2	71	27	17	0.6
Q9ULX6-2	A-kinase anchor protein 8-like	10	8	4	0.6
P49588	Alanine--tRNA ligase, cytoplasmic	419	58	41	0.7
P15121	Aldose reductase	166	76	17	0.7
P61163	Alpha-centractin	67	41	9	0.7
P40222	Alpha-taxilin	26	16	7	0.7
Q06203	Amidophosphoribosyltransferase	33	16	7	0.7
E5RJ99	AN1-type zinc finger protein 1	9	15	3	0.6
Q8IWZ3	Ankyrin repeat and KH domain-containing protein 1	29	4	6	0.5
H0YM23	Ankyrin repeat domain-containing protein 17	34	5	3	0.6
P08758	Annexin A5	226	67	19	0.7
Q9BXS5	AP-1 complex subunit mu-1	26	21	7	0.6
Q9ULZ3-3	Apoptosis-associated speck-like protein containing a CARD	26	32	4	0.5
P20292	Arachidonate 5-lipoxygenase-activating protein	16	19	4	0.6
P53367	Arfaptin-1	34	29	7	0.7
P04424-3	Argininosuccinatelyase	14	14	5	0.7
P00966	Argininosuccinate synthase	19	21	7	0.3
P00505	Aspartate aminotransferase, mitochondrial	377	63	26	0.6
Q15121	Astrocyticphosphoprotein PEA-15	46	45	6	0.5
P11912	B-cell antigen receptor complex-associated protein alpha chain	14	35	4	0.5
P13995	Bifunctionalmethylenetetrahydrofolate dehydrogenase	42	32	8	0.6
P54687-4	Branched-chain-amino-acid aminotransferase, cytosolic	98	31	12	0.7
Q8WY22	BRI3-binding protein	22	17	3	0.6
F6U1T9	Calcineurin subunit B type 1	28	26	3	0.6
Q9BRF8	Calcineurin-like phosphoesterase domain-containing protein 1	23	29	6	0.7

Q13557-12	Calcium/calmodulin-dependent protein kinase type II	59	27	11	0.7
Q9H9S4	Calcium-binding protein 39-like	79	44	11	0.5
E7EQ12	Calpastatin	15	8	2	0.6
O43852	Calumenin	136	47	13	0.7
Q9BXW7-2	Cat eye syndrome critical region protein 5	14	13	4	0.6
F5GXJ9	CD166 antigen	14	13	4	0.5
E9PNW4	CD59 glycoprotein	10	19	2	0.1
P32970	CD70 antigen	23	20	3	0.5
E9PJK1	CD81 antigen	78	41	4	0.5
A6NNI4	CD9 antigen	17	14	2	0.4
Q9Y696	Chloride intracellular channel protein 4	127	67	15	0.7
Q13185	Chromobox protein homolog 3	73	59	7	0.7
P45973	Chromobox protein homolog 5	45	37	7	0.4
Q7RTS1	Class A basic helix-loop-helix protein 15	30	30	4	0.6
Q14019	Coactosin-like protein	89	68	11	0.6
Q6P1N0-2	Coiled-coil and C2 domain-containing protein 1A	16	4	4	0.7
Q9H6F5	Coiled-coil domain-containing protein 86	10	17	4	0.6
Q9UP83-3	Conserved oligomeric Golgi complex subunit 5	16	8	4	0.7
O75367-2	Core histone macro-H2A.1	82	48	13	0.4
E9PKA1	CUGBP Elav-like family member 1	10	43	2	0.5
H0YAZ9	Cyclin-dependent kinase 16	17	6	1	0.5
P42773	Cyclin-dependent kinase 4 inhibitor C	25	26	3	0.7
Q00534	Cyclin-dependent kinase 6	74	40	9	0.7
E9PND2	Cysteine and glycine-rich protein 1	14	37	3	0.5
Q9Y4P1-6	Cysteine protease ATG4B	25	23	7	0.7
B7Z382	Cytosolic purine 5'-nucleotidase	19	17	6	0.6
O43175	D-3-phosphoglycerate dehydrogenase	272	52	27	0.5
C9JAB0	DCC-interacting protein 13-alpha	9	25	2	0.6
Q9NR28-2	Diablo homolog, mitochondrial	27	49	7	0.6
O76075-2	DNA fragmentation factor subunit beta	14	7	1	0.7
P49917	DNA ligase 4	9	5	2	0.7
P28340	DNA polymerase delta catalytic subunit	32	8	8	0.7
P11388	DNA topoisomerase 2-alpha	15	3	1	0.6
O14593	DNA-binding protein RFXANK	11	20	2	0.5
Q8WXX5	DnaJ homolog subfamily C member 9	17	12	3	0.7
Q14203-3	Dynactin subunit 1	113	23	22	0.7
F5H223	Dynactin subunit 2	26	27	8	0.6
O60313	Dynamin-like 120 kDa protein, mitochondrial	56	13	12	0.6
Q5VTU3	Dynein light chain Tctex-type 1	42	37	2	0.7
Q9Y4X5	E3 ubiquitin-protein ligase ARIH1	19	14	5	0.6
Q5T4S7-3	E3 ubiquitin-protein ligase UBR4	73	4	15	0.6
Q9HC35	Echinoderm microtubule-associated protein-like 4	10	5	4	0.6
Q14657	EKC/KEOPS complex subunit LAGE3	26	31	3	0.7

P29692-3	Elongation factor 1-delta	202	54	1	0.4
P49411	Elongation factor Tu, mitochondrial	208	50	21	0.5
Q96FZ2	Embryonic stem cell-specific protein	25	20	5	0.4
Q9BS26	Endoplasmic reticulum resident protein 44	77	35	11	0.7
P42126	Enoyl-CoA delta isomerase 1, mitochondrial	53	27	7	0.7
S4R3U1	Epidermal growth factor receptor substrate 15	12	46	1	0.4
Q53GQ0	Estradiol 17-beta-dehydrogenase 12	80	51	12	0.7
O43324	Eukaryotic translation elongation factor 1 epsilon-1	81	64	10	0.7
O60739	Eukaryotic translation initiation factor 1b	49	62	1	0.7
Q9Y5B9	FACT complex subunit SPT16	54	17	15	0.6
Q08945	FACT complex subunit SSRP1	48	22	14	0.5
Q86UX7-2	Fermitin family homolog 3	176	41	19	0.6
P02794	Ferritin heavy chain	35	42	6	0.2
Q9H488	GDP-fucose protein O-fucosyltransferase 1	19	13	3	0.6
Q3V6T2-5	Girdin	83	12	17	0.5
M0QYG8	Glia maturation factor gamma	12	31	2	0.7
O94925	Glutaminase kidney isoform, mitochondrial	46	20	1	0.7
P35754	Glutaredoxin-1	46	71	5	0.6
Q86SX6	Glutaredoxin-related protein 5, mitochondrial	21	21	4	0.5
P78417	Glutathione S-transferase omega-1	33	39	10	0.6
P09211	Glutathione S-transferase P	500	64	12	0.7
P48637	Glutathione synthetase	17	14	8	0.6
P35573-2	Glycogen debranching enzyme	24	7	9	0.6
P11216	Glycogen phosphorylase, brain form	65	22	15	0.7
Q92896	Golgi apparatus protein 1	69	16	14	0.3
H7BXD5	Grancalcin	28	50	8	0.6
F8W9S7	GTPase-activating protein and VPS9 domain-containing protein	72	14	14	0.7
Q14344	Guanine nucleotide-binding protein subunit alpha-13	88	46	13	0.7
Q9BVP2-2	Guanine nucleotide-binding protein-like 3	59	17	8	0.6
H7C0M1	H/ACA ribonucleoprotein complex subunit 4	10	24	3	0.6
O00165-5	HCLS1-associated protein X-1	24	35	5	0.6
Q7Z4H3-2	HD domain-containing protein 2	15	35	5	0.6
P14317	Hematopoietic lineage cell-specific protein	187	44	18	0.7
Q7LGA3	Heparan sulfate 2-O-sulfotransferase 1	12	5	1	0.4
O14964-2	Hepatocyte growth factor-regulated tyrosine kinase substrate	10	4	2	0.5
P55795	Heterogeneous nuclear ribonucleoprotein H2	142	21	1	0.6
Q00839	Heterogeneous nuclear ribonucleoprotein U	328	37	1	0.6
P17096-2	High mobility group protein HMG-I/HMG-Y	11	31	1	0.7
P17096	High mobility group protein HMG-I/HMG-Y	23	38	3	0.5
P49773	Histidine triad nucleotide-binding protein 1	108	74	6	0.7
P16403	Histone H1.2	112	33	3	0.4
P16402	Histone H1.3	104	39	6	0.5

P10412	Histone H1.4	114	37	3	0.5
P16401	Histone H1.5	88	31	10	0.4
Q92522	Histone H1x	34	33	7	0.5
P04908	Histone H2A type 1-B/E	157	41	1	0.2
Q6FI13	Histone H2A type 2-A	188	63	2	0.4
Q8IUE6	Histone H2A type 2-B	76	42	4	0.5
P0C0S5	Histone H2A.Z	92	54	4	0.5
O60814	Histone H2B type 1-K	157	56	4	0.3
Q8N257	Histone H2B type 3-B	149	50	3	0.4
K7EK07	Histone H3	52	45	1	0.3
P68431	Histone H3.1	61	51	1	0.3
Q71DI3	Histone H3.2	72	51	1	0.3
P62805	Histone H4	201	54	10	0.4
Q6YN16	Hydroxysteroid dehydrogenase-like protein 2	15	15	4	0.6
D6RD17	Immunoglobulin J chain	10	28	4	0.6
Q969P0-3	Immunoglobulin superfamily member 8	22	14	5	0.6
O15111	Inhibitor of nuclear factor kappa-B kinase subunit alpha	14	2	1	0.4
Q9NZI8	Insulin-like growth factor 2 mRNA-binding protein 1	175	42	19	0.6
Q27J81-2	Inverted formin-2	15	2	2	0.5
P50213	Isocitrate dehydrogenase [NAD] subunit alpha, mitochondrial	65	36	11	0.5
O43837	Isocitrate dehydrogenase [NAD] subunit beta, mitochondrial	38	23	8	0.4
E9PF84	Isocitrate dehydrogenase [NAD] subunit gamma, mitochondrial	11	22	4	0.3
Q96BW5-2	Isoform 2 of Phosphotriesterase-related protein	54	44	11	0.5
Q9NY93-2	Isoform 2 of Probable ATP-dependent RNA helicase DDX56	21	14	5	0.6
Q9GZT8-2	Isoform 2 of Putative GTP cyclohydrolase 1 type 2 NIF3L1	11	10	3	0.6
Q4G0N4-3	Isoform 3 of NAD kinase 2, mitochondrial	31	35	7	0.4
Q8N983-4	Isoform 4 of 39S ribosomal protein L43, mitochondrial	23	37	6	0.4
P14618-2	Isoform M1 of Pyruvate kinase PKM	1141	69	4	0.7
P60660-2	Isoform Smooth muscle of Myosin light polypeptide 6	89	76	8	0.6
O00139-2	Kinesin-like protein KIF2A	35	12	8	0.6
Q01650	Large neutral amino acids transporter small subunit 1	15	9	3	0.7
Q9UIQ6-3	Leucyl-cystinylaminopeptidase	120	25	26	0.6
Q08722-2	Leukocyte surface antigen CD47	9	10	3	0.5
P19397	Leukocyte surface antigen CD53	14	7	2	0.3
C9JUK7	Leukosialin	16	8	3	0.4
Q14847	LIM and SH3 domain protein 1	40	48	11	0.7
F8VRN8	LIM domain and actin-binding protein 1	10	3	1	0.5
J3QQM5	LIM domain-containing protein 2	10	27	3	0.6
Q9GZY6	Linker for activation of T-cells family member 2	10	16	2	0.5
O95573	Long-chain-fatty-acid--CoA ligase 3	14	8	2	0.7
Q12912-2	Lymphoid-restricted membrane protein	144	48	24	0.6

H7C413	Lys-63-specific deubiquitinase BRCC36	9	35	1	0.7
P10619	Lysosomal protective protein	11	5	2	0.3
Q8WU39	Marginal zone B- and B1-cell-specific protein	60	39	5	0.7
Q9NZD8-2	Maspardin	14	14	4	0.6
B7Z9I1	Medium-chain-specific acyl-CoA dehydrogenase	102	40	13	0.6
P53582	Methionine aminopeptidase 1	52	29	8	0.5
O14880	Microsomal glutathione S-transferase 3	22	18	3	0.6
Q7Z434	Mitochondrial antiviral-signaling protein	30	9	3	0.7
O43615	Mitochondrial import inner membrane translocase	122	38	19	0.7
Q7L0Y3	Mitochondrial ribonuclease P protein 1	30	25	9	0.6
P27361	Mitogen-activated protein kinase 3	32	26	5	0.7
Q9BU76-4	Multiple myeloma tumor-associated protein 2	10	19	3	0.7
Q9NUJ1	Mycophenolic acid acyl-glucuronide esterase, mitochondrial	75	45	10	0.6
P19105	Myosin regulatory light chain 12A	58	57	7	0.6
P13533	Myosin-6	9	1	1	0.1
P35579	Myosin-9	1293	57	99	0.6
Q86U44	N6-adenosine-methyltransferase 70 kDa subunit	19	15	7	0.6
P56556	NADH dehydrogenase [ubiquinone] 1 alpha subcomplex	24	29	4	0.4
O95299	NADH dehydrogenase [ubiquinone] 1 alpha subcomplex	37	26	4	0.3
Q9UI09	NADH dehydrogenase [ubiquinone] 1 alpha subcomplex	10	23	3	0.4
Q16795	NADH dehydrogenase [ubiquinone] 1 alpha subcomplex	50	31	8	0.7
P17568	NADH dehydrogenase [ubiquinone] 1 beta subcomplex	10	14	2	0.7
P49821-2	NADH dehydrogenase [ubiquinone] flavoprotein 1	30	20	8	0.5
P19404	NADH dehydrogenase [ubiquinone] flavoprotein 2	16	21	4	0.4
P56181-2	NADH dehydrogenase [ubiquinone] flavoprotein 3	11	7	3	0.5
O75489	NADH dehydrogenase [ubiquinone] iron-sulfur protein 3	106	52	14	0.7
O43920	NADH dehydrogenase [ubiquinone] iron-sulfur protein 5	26	51	5	0.7
O00217	NADH dehydrogenase [ubiquinone] iron-sulfur protein 8	17	34	5	0.6
P28331-4	NADH-ubiquinone oxidoreductase	95	32	14	0.4
B4DR03	N-alpha-acetyltransferase 40	10	13	2	0.6
Q9Y5A7-2	NEDD8 ultimate buster 1	29	16	7	0.6
Q13564-2	NEDD8-activating enzyme E1 regulatory subunit	22	18	6	0.6
P08473	Neprilysin	48	24	15	0.6
Q15758	Neutral amino acid transporter B(0)	82	22	9	0.7
P05114	Non-histone chromosomal protein HMG-14	10	31	3	0.5
P61970	Nuclear transport factor 2	17	57	4	0.6
O00567	Nucleolar protein 56	97	39	20	0.5
Q9Y2X3	Nucleolar protein 58	93	33	16	0.6
Q9H6R4-4	Nucleolar protein 6	45	11	8	0.5
Q9UMY1	Nucleolar protein 7	10	13	2	0.7
P15531	Nucleoside diphosphate kinase A	187	86	2	0.5
Q9BQ69	O-acetyl-ADP-ribose deacetylase MACROD1	34	18	4	0.4
Q92882	Osteoclast-stimulating factor 1	27	34	6	0.7

P50897	Palmitoyl-protein thioesterase 1	17	25	4	0.4
Q96EY7	Pentatricopeptide repeat domain-containing protein	18	11	4	0.2
Q00688	Peptidyl-prolylcis-trans isomerase FKBP3	56	38	9	0.7
J3KS15	Peptidyl-tRNA hydrolase ICT1, mitochondrial	14	23	4	0.3
Q13162	Peroxiredoxin-4	180	65	13	0.7
Q8WW12	PEST proteolytic signal-containing nuclear protein	40	48	8	0.7
O15357	Phosphatidylinositol 3,4,5-trisphosphate 5-phosphatase 2	29	7	7	0.7
Q16822	Phosphoenolpyruvatecarboxykinase [GTP], mitochondrial	55	22	12	0.6
Q9NWQ8	Phosphoprotein associated with glycosphingolipid-enriched	11	8	2	0.3
Q14558	Phosphoribosyl pyrophosphate synthase-associated protein 1	85	44	9	0.7
Q9Y617	Phosphoserine aminotransferase	299	68	18	0.4
C9JBI3	Phosphoserine phosphatase	83	63	9	0.4
Q6NYC8	Phostensin O	25	12	5	0.6
O15031	Plexin-B2	34	7	9	0.7
P09874	Poly [ADP-ribose] polymerase 1	382	43	44	0.4
Q9BY77	Polymerase delta-interacting protein 3	28	20	6	0.7
P14859-4	POU domain, class 2, transcription factor 1	18	5	2	0.5
Q8IY81	pre-rRNA processing protein FTSJ3	50	17	10	0.6
Q92841	Probable ATP-dependent RNA helicase DDX17	185	42	22	0.6
Q96GQ7	Probable ATP-dependent RNA helicase DDX27	23	10	7	0.7
Q8NBJ5	Procollagengalactosyltransferase 1	13	6	4	0.4
Q9BUL8	Programmed cell death protein 10	38	31	7	0.7
Q16186	Proteasomal ubiquitin receptor ADRM1	14	11	3	0.5
P28074	Proteasome subunit beta type-5	98	41	12	0.6
K7EK88	Protein AATF	10	26	2	0.4
Q13015	Protein AF1q	10	48	3	0.4
O75629	Protein CREG1	44	18	3	0.4
P35659	Protein DEK	58	27	12	0.7
C9J6N5	Protein FAM107B	41	37	6	0.6
Q96C01	Protein FAM136A	10	20	2	0.3
Q92520	Protein FAM3C	33	36	8	0.6
E9PH82	Protein FAM98A	24	13	2	0.7
Q9UNF0-2	Protein kinase C and casein kinase substrate in neurons protein	26	17	7	0.5
G3V5L1	Protein max	10	25	2	0.5
Q9UFN0	Protein NipSnap homolog 3A	62	44	8	0.6
Q8NBL1	Protein O-glucosyltransferase 1	10	8	3	0.5
Q15435	Protein phosphatase 1 regulatory subunit 7	62	37	10	0.7
Q14690	Protein RRP5 homolog	35	6	9	0.6
P31949	Protein S100-A11	18	50	5	0.6
A6NIH7	Protein unc-119 homolog B	17	22	4	0.6
P22061	Protein-L-isoaspartate(D-aspartate) O-methyltransferase	84	62	10	0.6
Q16740	Putative ATP-dependent Clp protease proteolytic subunit	39	30	6	0.7

P32322	Pyrroline-5-carboxylate reductase 1, mitochondrial	40	30	5	0.4
Q96C36	Pyrroline-5-carboxylate reductase 2	18	21	3	0.5
P08559	Pyruvate dehydrogenase E1 component subunit alpha	24	18	6	0.7
P31150	Rab GDP dissociation inhibitor alpha	177	23	1	0.6
P52306	Rap1 GTPase-GDP dissociation stimulator 1	48	33	14	0.7
Q8WUD1	Ras-related protein Rab-2B	52	36	2	0.7
F5H157	Ras-related protein Rab-35	45	27	2	0.6
P51151	Ras-related protein Rab-9A	9	19	3	0.7
P11234	Ras-related protein Ral-B	30	43	3	0.6
P61225	Ras-related protein Rap-2b	21	13	1	0.6
Q9Y3L5	Ras-related protein Rap-2c	16	17	1	0.5
P08575-2	Receptor-type tyrosine-protein phosphatase C	118	24	22	0.7
P35251-2	Replication factor C subunit 1	22	6	5	0.6
O95197-3	Reticulon-3	29	9	2	0.5
Q15291-2	Retinoblastoma-binding protein 5	14	4	2	0.6
Q9HB40	Retinoid-inducible serine carboxypeptidase	23	12	5	0.4
P49247	Ribose-5-phosphate isomerase	106	54	15	0.5
Q15050	Ribosome biogenesis regulatory protein homolog	31	30	9	0.5
Q9Y3A5	Ribosome maturation protein SBDS	76	52	14	0.7
Q9H7B2	Ribosome production factor 2 homolog	42	26	7	0.5
O00442	RNA 3'-terminal phosphate cyclase	27	19	6	0.7
Q92541	RNA polymerase-associated protein RTF1 homolog	10	6	4	0.3
Q9NW13	RNA-binding protein 28	60	14	8	0.6
Q96DH6-2	RNA-binding protein Musashi homolog 2	28	26	5	0.7
Q96T51-2	RUN and FYVE domain-containing protein 1	13	6	3	0.6
O15126	Secretory carrier-associated membrane protein 1	27	14	3	0.6
D6RGI3	Septin 11, isoform CRA_b	69	43	8	0.6
B4DR80	Serine/threonine-protein kinase 24 12 kDa subunit	34	24	4	0.7
Q8NBY1	Serine/threonine-protein kinase MST4	40	39	7	0.7
H0YDU8	Serine/threonine-protein phosphatase	44	20	10	0.7
Q08209-3	Serine/threonine-protein phosphatase 2B catalytic	20	14	6	0.7
Q8TF05-2	Serine/threonine-protein phosphatase 4 regulatory subunit 1	19	4	3	0.6
P49591	Serine--tRNA ligase, cytoplasmic	105	43	18	0.7
P50453	Serpin B9	22	15	5	0.5
P10768	S-formylglutathione hydrolase	154	76	15	0.7
H0YNG3	Signal peptidase complex catalytic subunit SEC11A	20	21	5	0.7
P05023	Sodium/potassium-transporting ATPase subunit alpha-1	182	35	19	0.6
G3XAL9	Sodium/potassium/chloride transporter	11	2	1	0.7
Q96L92-2	Sorting nexin-27	12	6	3	0.6
P52788	Spermine synthase	60	45	11	0.6
P31948	Stress-induced-phosphoprotein 1	277	49	34	0.7
Q99470	Stromal cell-derived factor 2	32	23	3	0.6
Q8WU79-2	Stromal membrane-associated protein 2	11	8	3	0.5

Q6ZRP7	Sulfhydryl oxidase 2	28	12	6	0.4
P00441	Superoxide dismutase [Cu-Zn]	246	86	10	0.7
P04179-4	Superoxide dismutase [Mn], mitochondrial	96	60	9	0.7
Q9UH65	Switch-associated protein 70	145	46	23	0.7
Q96A49	Synapse-associated protein 1	25	21	4	0.6
O00186	Syntaxin-binding protein 3	24	13	6	0.7
E7EPD0	Target of Myb protein 1	10	8	3	0.6
P51580	Thiopurine S-methyltransferase	9	12	3	0.6
H7BZJ3	Thioredoxin	67	58	1	0.7
B7Z2S5	Thioredoxinreductase 1, cytoplasmic	110	47	20	0.7
Q96CG3	TRAF-interacting protein with FHA domain-containing protein	23	43	7	0.6
Q14241	Transcription elongation factor B polypeptide 3	9	4	2	0.6
Q00059	Transcription factor A, mitochondrial	22	29	8	0.4
Q9UI10-3	Translation initiation factor eIF-2B subunit delta	26	19	6	0.6
P40939	Trifunctional enzyme subunit alpha, mitochondrial	137	39	20	0.6
P55084	Trifunctional enzyme subunit beta, mitochondrial	60	25	11	0.5
P23381	Tryptophan--tRNA ligase, cytoplasmic	253	67	25	0.6
Q9Y3Q8	TSC22 domain family protein 4	12	21	4	0.6
Q13509	Tubulin beta-3 chain	450	65	6	0.7
Q9UBB9	Tuftelin-interacting protein 11	9	5	3	0.6
O43399	Tumor protein D54	71	51	2	0.7
Q9UIG0-2	Tyrosine-protein kinase BAZ1B	26	6	7	0.6
P06241	Tyrosine-protein kinase Fyn	19	4	1	0.7
P43405	Tyrosine-protein kinase SYK	16	15	5	0.7
P54577	Tyrosine--tRNA ligase, cytoplasmic	196	63	34	0.6
Q9BVJ6	U3 small nucleolar RNA-associated protein 14 homolog A	17	9	5	0.6
Q9Y5J1	U3 small nucleolar RNA-associated protein 18 homolog	11	12	4	0.7
O15116	U6 snRNA-associated Sm-like protein LSm1	23	42	4	0.7
Q9NRR5	Ubiquilin-4	42	14	3	0.6
P09936	Ubiquitin carboxyl-terminal hydrolase isozyme L1	283	83	15	0.7
A6NJ11	Ubiquitin fusion degradation protein 1 homolog	17	23	4	0.6
P51668	Ubiquitin-conjugating enzyme E2 D1	32	24	2	0.6
Q5QPP3	UDP-glucose 4-epimerase	10	12	2	0.5
F8W031	Uncharacterized protein	51	30	6	0.7
Q9BUH6	Uncharacterized protein C9orf142	14	23	3	0.6
Q8IYS2	Uncharacterized protein KIAA2013	23	11	5	0.6
Q16851	UTP--glucose-1-phosphate uridylyltransferase	51	28	13	0.7
P49748	Very long-chain specific acyl-CoA dehydrogenase,	73	28	14	0.6
Q8WV19	Vesicle transport protein SFT2A	13	17	1	0.3
Q5TIH2	Vesicle transport protein SFT2B	13	25	2	0.7
Q587I9	Vesicle transport protein SFT2C	15	20	3	0.4
O75379-2	Vesicle-associated membrane protein 4	10	24	3	0.5

Q13303-4	Voltage-gated potassium channel subunit beta-2	40	26	8	0.6
O75083	WD repeat-containing protein 1	132	42	17	0.6
Q15061	WD repeat-containing protein 43	14	5	3	0.6
Q5SZK5	Wiskott-Aldrich syndrome protein family member 1	13	18	1	0.2
F8W840	YTH domain-containing family protein 1	28	12	2	0.5
R4GN55	YTH domain-containing family protein 3	33	15	3	0.4
Q7Z2W4	Zinc finger CCCH-type antiviral protein 1	24	8	5	0.4
