# Supplementary material for: Proteomic Analysis of Gossypol Induces Necrosis in Multiple Myeloma Cells
Source: Biomed Res Int. 2014 Aug 14;2014:839232. doi: 10.1155/2014/839232 (PMC4150408; doi:10.1155/2014/839232)
Supplement: Supplementary file 1 — Supplementary Figure 1. Gossypol induced the fold of changes in DNA damage repair and replication associated proteins from the untreated and 40 μM gossypol-treated U266 cells for 24 h as determined by quantitative proteomics. Supplementary Figure 2. Gossypol induced the fold of changes in mitochondrial 28S associated proteins from the untreated and 40 μM gossypol-treated U266 cells for 24 h as determined by quantitative proteomics. Supplementary Figures 3. Gossypol induced the fold of changes in mitochondrial 39S associated proteins from the untreated and 40 μM gossypol-treated U266 cells for 24 h as determined by quantitative proteomics. Supplementary Figure 4. The MS/MS spectra of peptides for identification of selected proteins. Supplementary Table 1. Up-regulated proteins in gossypol-treated cells. Supplementary Table 2. Down-regulated proteins in gossypol-treated cells. [file 839232.f1.zip › Supplementary Table1(RHX).pdf]

Supplementary Table 1. Up-regulated proteins in gossypol-treated cells

Accession	Description	Score	Coverage	Unique Peptides	Gossypol / Control
Q04446	1,4-alpha-glucan-branching enzyme	50	15	10	1.8
Q8N543	2-oxoglutarate and iron-dependent oxygenase protein 1	37	17	8	1.9
H7C131	3-ketoacyl-CoA thiolase, peroxisomal	21	28	6	1.7
Q5TFE4	5'-nucleotidase domain-containing protein 1	40	36	9	2.2
P10809	60 kDa heat shock protein, mitochondrial	430	60	25	1.9
P53999	Activated RNA polymerase II transcriptional coactivator p15	38	39	7	5.2
P27144	Adenylate kinase 4, mitochondrial	49	61	10	2.1
E9PQP3	ADP-ribosylation factor GTPase-activating protein 2	10	15	2	1.8
P35611-2	Alpha-adducin	20	10	4	1.8
Q9Y679-2	Ancient ubiquitous protein 1	19	15	5	3.4
F5H648	Antigen peptide transporter 1	11	11	3	2.3
Q8IUR7-3	Armadillo repeat-containing protein 8	30	12	4	1.8
Q8IYB8	ATP-dependent RNA helicase SUPV3L1, mitochondrial	20	8	5	1.9
Q99856	AT-rich interactive domain-containing protein 3A	14	13	3	7.6
Q96BJ3	Axininteractor, dorsalization-associated protein	13	18	3	1.6
Q16611	Bcl-2 homologous antagonist/killer	9	11	2	2.0
P61769	Beta-2-microglobulin	33	49	6	2.6
Q562R1	Beta-actin-like protein 2	193	19	1	3.8
P25098	Beta-adrenergic receptor kinase 1	39	16	8	1.9
B7Z9G5	BRO1 domain-containing protein BROX	20	15	3	2.2
O60885	Bromodomain-containing protein 4	32	6	4	1.7
Q99653	Calcineurin B homologous protein 1	27	46	7	1.6
P09326	CD48 antigen	9	13	3	1.7
P60953-1	Cell division control protein 42 homolog	165	50	1	2.6
Q9BYV8	Centrosomal protein of 41 kDa	11	12	3	2.0
Q8WVB6	Chromosome transmission fidelity protein 18 homolog	12	6	4	1.7
Q10570	Cleavage and polyadenylation specificity factor subunit 1	11	3	3	2.2
Q9H0L4	Cleavage stimulation factor subunit 2 tau variant	12	11	3	1.7
Q12996	Cleavage stimulation factor subunit 3	25	12	6	1.7
Q99417	C-Myc-binding protein	13	65	5	1.8
P12259	Coagulation factor V	60	6	14	8.0
B8ZZ99	Coiled-coil domain-containing protein 115	13	15	2	1.9
Q9H6E4	Coiled-coil domain-containing protein 134	16	22	4	2.2
Q8N668	COMM domain-containing protein 1	16	26	3	1.6
Q9UBI1	COMM domain-containing protein 3	39	36	2	1.8
P42695	Condensin-2 complex subunit D3	24	7	7	2.2
Q9BZJ0-2	Crooked neck-like protein 1	20	10	6	2.1

O75909	Cyclin-K	9	9	3	2.1
Q15438-2	Cytohesin-1	27	15	5	1.9
E9PCG9	D-beta-hydroxybutyrate dehydrogenase, mitochondrial	12	17	3	1.8
G3V3G9	DDB1- and CUL4-associated factor 8	10	3	1	2.0
Q96BY6-3	Dedicator of cytokinesis protein 10	14	3	4	1.8
P54886-2	Delta-1-pyrroline-5-carboxylate synthase	224	52	32	1.6
P32321	Deoxycytidylatedeaminase	17	21	3	2.4
Q9BU89	Deoxyhypusine hydroxylase	21	26	4	3.1
P49366	Deoxyhypusine synthase	30	23	5	1.9
P33316	Deoxyuridine 5'-triphosphate nucleotidohydrolase	150	54	11	1.8
P00374	Dihydrofolatereductase	27	43	8	1.9
P26358	DNA (cytosine-5)-methyltransferase 1	92	16	22	2.0
B4DTU4	DNA ligase	77	26	18	2.9
H0Y818	DNA mismatch repair protein Mlh1	21	12	5	2.1
Q14691	DNA replication complex GINS protein PSF1	16	30	4	2.2
Q9BRX5	DNA replication complex GINS protein PSF3	25	35	6	2.5
E5RFF9	DNA replication complex GINS protein SLD5	13	17	2	3.7
P31689	DnaJ homolog subfamily A member 1	115	51	15	1.7
Q13217	DnaJ homolog subfamily C member 3	14	8	3	1.7
Q5VTR2	E3 ubiquitin-protein ligase BRE1A	31	9	6	1.7
O75150-4	E3 ubiquitin-protein ligase BRE1B	43	15	10	1.7
Q7Z6E9-2	E3 ubiquitin-protein ligase RBBP6	21	3	5	1.7
Q14669-4	E3 ubiquitin-protein ligase TRIP12	22	6	7	1.8
Q9NZN3	EH domain-containing protein 3	42	17	1	1.8
Q9H223	EH domain-containing protein 4	23	13	4	1.8
P29692-4	Elongation factor 1-delta	170	50	1	2.0
B4DKB2	Endothelin-converting enzyme 1	14	7	3	2.6
O60344-4	Endothelin-converting enzyme 2	15	21	3	1.7
Q8N766-2	ER membrane protein complex subunit 1	9	4	3	3.8
Q9HAV4	Exportin-5	67	12	11	2.4
P11166	Facilitated glucose transporter member 1	18	4	3	1.7
Q9NVI1	Fanconi anemia group I protein	87	22	21	1.9
P09972	Fructose-bisphosphatealdolase C	180	55	8	2.7
Q96GK7	Fumarylacetoacetate hydrolase domain-containing protein 2A	11	18	3	2.1
P13284	Gamma-interferon-inducible lysosomalthiolreductase	17	23	3	3.1
Q03013-2	Glutathione S-transferase Mu 4	31	51	6	1.9
Q08379	Golgin subfamily A member 2	35	10	7	1.6
H0YK03	Golgin subfamily A member 5	9	21	2	1.6
Q12849	G-rich sequence factor 1	24	20	5	10.5
O00178	GTP-binding protein 1	11	12	4	2.0
Q9HAV0	Guanine nucleotide-binding protein subunit beta-4	30	27	2	3.7
P08238	Heat shock protein HSP 90-beta	3035	73	37	1.6

P54652	Heat shock-related 70 kDa protein 2	444	23	2	3.8
Q14527-2	Helicase-like transcription factor	13	5	3	2.6
J3KT51	Hematological and neurological-expressed 1 protein	30	56	5	1.7
G3V2Q1	Heterogeneous nuclear ribonucleoproteins C1/C2	248	51	1	3.8
E9PB90	Hexokinase-2	128	28	17	2.6
P01891	HLA class I histocompatibility antigen, A-68 alpha chain	133	39	2	2.3
Q5SRN7	HLA class I histocompatibility antigen, A-68 alpha chain	163	58	7	1.7
P30487	HLA class I histocompatibility antigen, B-49 alpha chain	196	47	3	2.3
P04233-2	HLA class II histocompatibility antigen gamma chain	50	31	7	4.4
H0Y6F8	HLA class II histocompatibility antigen, DP alpha 1 chain	37	23	2	2.0
A2AAZ0	HLA class II histocompatibility antigen, DQ beta 1 chain	19	29	6	4.0
Q30154	HLA class II histocompatibility antigen, DR beta 5 chain	138	39	2	1.7
P39880-4	Homeobox protein cut-like 1	53	14	5	1.8
P52292	Importin subunit alpha-1	30	19	6	1.9
Q9UI26	Importin-11	43	13	8	2.7
Q6P9B9	Integrator complex subunit 5	12	5	3	1.9
Q8IU81	Interferon regulatory factor 2-binding protein 1	10	8	3	1.7
E7EPJ2	Intersectin-2	14	9	4	1.9
P55196-2	Isoform 1 of Afadin	10	2	2	2.2
Q86UK7-2	Isoform 2 of Zinc finger protein 598	9	5	3	1.8
P29728-2	Isoform p69 of 2'-5'-oligoadenylate synthase 2	21	12	7	2.3
Q9H0B6	Kinesin light chain 2	25	13	2	1.8
P52732	Kinesin-like protein KIF11	68	22	15	1.7
B7Z6Q6	Kinesin-like protein KIF2C	14	7	4	2.8
Q9BW19	Kinesin-like protein KIFC1	26	13	6	1.6
Q8NBT2	Kinetochore protein Spc24	40	49	5	1.6
Q9HBM1	Kinetochore protein Spc25	20	28	4	2.0
P48449-2	Lanosterol synthase	10	7	5	1.7
O95232	Luc7-like protein 3	47	23	7	1.9
Q9Y4C1	Lysine-specific demethylase 3A	24	8	7	3.3
Q7LBC6	Lysine-specific demethylase 3B	35	10	10	2.0
Q13571	Lysosomal-associated transmembrane protein 5	9	9	2	2.9
G3V267	Maleylacetoacetateisomerase	29	34	4	1.7
Q96IJ6	Mannose-1-phosphate guanyltransferase alpha	11	8	3	3.3
Q9H204	Mediator of RNA polymerase II transcription subunit 28	9	20	2	2.5
G3XA91	Methionine aminopeptidase 2	123	57	2	2.1
H3BTL1	Microtubule-associated protein 1 light chain 3 beta	10	37	3	2.7
Q9BTE3-3	Mini-chromosome maintenance complex-binding protein	17	12	4	4.1
Q9Y3D6	Mitochondrial fission 1 protein	21	24	4	1.9
O43148	mRNA cap guanine-N7 methyltransferase	20	19	7	1.6
O95671-3	N-acetylserotonin O-methyltransferase-like protein	14	11	3	2.7
Q9UBB6-2	Neurochondrin	29	13	7	1.8

M0R3B2	Nitric oxide synthase-interacting protein	29	38	6	1.7
O00746	Nucleoside diphosphate kinase, mitochondrial	10	19	3	2.2
O00151	PDZ and LIM domain protein 1	52	40	8	3.0
Q9H2J4	Phosducin-like protein 3	18	21	5	1.9
Q8NC51	Plasminogen activator inhibitor 1 RNA-binding protein	114	45	2	1.7
Q8NC51-3	Plasminogen activator inhibitor 1 RNA-binding protein	137	53	4	2.1
Q9NX46	Poly(ADP-ribose) glycohydrolase ARH3	10	15	3	2.4
Q15366-6	Poly(rC)-binding protein 2	199	55	1	1.9
Q6UN15-5	Pre-mRNA 3'-end-processing factor FIP1	54	24	8	1.7
Q8NAV1	Pre-mRNA-splicing factor 38A	16	26	6	1.7
Q2NL82	Pre-rRNA-processing protein TSR1 homolog	27	14	8	1.6
J3KTA4	Probable ATP-dependent RNA helicase DDX5	178	42	19	1.6
P07602	Prosaposin	59	21	10	1.6
J3QQN1	Proteasome subunit beta type-10	15	22	2	2.1
Q13948-10	Protein CASP	33	19	1	1.8
Q8TCG1-2	Protein CIP2A	11	4	2	2.0
Q9NSV4-1	Protein diaphanous homolog 3	14	5	4	2.2
F8W7Q4	Protein FAM162A	14	19	4	1.6
H3BUL4	Protein FAM192A	10	14	2	1.7
Q96GA3	Protein LTV1 homolog	19	11	4	2.0
Q9BPW8	Protein NipSnap homolog 1	51	37	8	2.0
P48634-2	Protein PRRC2A	27	7	9	2.0
Q9Y520-3	Protein PRRC2C	91	13	23	2.0
Q14160-2	Protein scribble homolog	11	3	3	1.9
Q01105	Protein SET	182	43	2	1.7
P18583-6	Protein SON	37	6	9	1.9
Q9UNS1-2	Protein timeless homolog	10	3	3	1.7
J3KNL6	Protein transport protein Sec16A	58	10	14	1.9
O95486	Protein transport protein Sec24A	23	7	6	1.8
O94855	Protein transport protein Sec24D	21	12	7	1.7
P61619	Protein transport protein Sec61 subunit alpha isoform 1	18	13	4	1.8
Q5T1J5	Putative protein CHCHD2P9	17	27	2	7.0
O60518	Ran-binding protein 6	36	6	1	2.0
P15153	Ras-related C3 botulinum toxin substrate 2	78	41	4	1.7
B7Z9I3	Renin receptor	33	29	6	2.6
Q86TG7	Retrotransposon-derived protein PEG10	10	7	3	2.2
Q68EM7-2	Rho GTPase-activating protein 17	17	9	4	2.0
Q15052-2	Rho guanine nucleotide exchange factor 6	13	8	2	1.7
H0YEF3	Ribonuclease H2 subunit C	19	27	4	1.8
P23921	Ribonucleoside-diphosphatereductase large subunit	129	38	22	3.5
O43709	Ribosome biogenesis methyltransferase WBSCR22	12	11	2	1.8
Q9P2E9-3	Ribosome-binding protein 1	58	25	16	2.0
Q9Y508-2	RING finger protein 114	13	30	5	1.6

Q6PD62	RNA polymerase-associated protein CTR9 homolog	51	16	15	1.8
Q9P2N5	RNA-binding protein 27	29	11	6	2.4
Q9ULX3	RNA-binding protein NOB1	14	19	4	2.6
E9PJB8	Selenoprotein S	19	23	5	1.7
E7EMC7	Sequestosome-1	21	20	4	19.5
Q9UQ35	Serine/arginine repetitive matrix protein 2	74	11	19	1.9
Q5T760	Serine/arginine-rich-splicing factor 11	41	19	5	1.7
F5H2U2	Serine/threonine-protein kinase PRP4 homolog	12	5	4	1.7
Q13362-2	Serine/threonine-protein phosphatase 2A 56 kDa regulatory	33	23	2	2.3
Q6IN85-2	Serine/threonine-protein phosphatase 4 regulatory subunit 3A	33	12	8	2.4
P42224	Signal transducer and activator of transcription 1-alpha/beta	50	19	10	2.5
A9IYQ1	Sister chromatid cohesion protein PDS5 homolog B	38	27	1	2.9
Q01082	Spectrin beta chain, non-erythrocytic 1	489	39	6	1.9
B3KQ95	Squalene synthase	14	20	5	18.6
Q92922	SWI/SNF complex subunit SMARCC1	69	18	8	1.7
O43752	Syntaxin-6	15	16	2	1.7
O00560-2	Syntenin-1	13	24	3	1.9
Q86VP1-2	Tax1-binding protein 1	9	5	4	1.9
Q5UIP0-2	Telomere-associated protein RIF1	66	11	17	2.6
P04183	Thymidine kinase, cytosolic	20	20	4	3.9
P04818-2	Thymidylate synthase	47	35	8	3.6
C9JER1	Thyroid adenoma-associated protein	13	5	4	2.0
P20290	Transcription factor BTF3	129	61	1	4.1
Q00403	Transcription initiation factor IIB	11	12	3	1.6
P29084	Transcription initiation factor IIE subunit beta	14	24	5	1.8
Q8WXI9	Transcriptional repressor p66-beta	39	18	6	1.9
P02786	Transferrin receptor protein 1	280	39	26	1.7
K7EQG9	Tyrosine-protein phosphatase non-receptor type	13	13	3	2.9
P29350	Tyrosine-protein phosphatase non-receptor type 6	47	26	10	4.0
O14933	Ubiquitin/ISG15-conjugating enzyme E2 L6	30	44	4	1.6
C9J2P0	Ubiquitin-conjugating enzyme E2 E1	12	18	1	1.8
P62253	Ubiquitin-conjugating enzyme E2 G1	12	33	3	2.4
Q92575	UBX domain-containing protein 4	15	19	5	1.9
Q9BZX2	Uridine-cytidine kinase 2	25	30	5	1.6
P06132	Uroporphyrinogen decarboxylase	20	15	3	2.1
Q9Y2W2	WW domain-binding protein 11	49	13	9	1.7
M0QY97	Zinc finger CCCH domain-containing protein 4	26	14	7	2.7
B4DT58	Zinc finger protein ubi-d4	9	15	2	2.0
